# *N*-(2-Meth­oxy­phen­yl)phthalamic acid

**DOI:** 10.1107/S1600536813013408

**Published:** 2013-05-22

**Authors:** Paul G. Waddell, Rupert J. Rutledge, Jacqueline M. Cole

**Affiliations:** a Cavendish Laboratory, University of Cambridge, J. J. Thomson Avenue, Cambridge CB3 0HE, England

## Abstract

The title compound, C_14_H_13_NO_3_, adopts a twisted conformation in the crystal, with an inter­planar angle between the two benzene rings of 87.30 (5)°. Mol­ecules within the structure are linked *via* O—H⋯O inter­actions, forming a hydrogen-bonded chain motif with graph set *C*(7) along the glide plane in the [001] direction.

## Related literature

For related phthalamic acid structures, see: Smith *et al.* (1983 ▶) and Bocelli *et al.* (1989 ▶). For the structure of a diaryl amide containing the 2-meth­oxy­phenyl moeity, see: Parra *et al.* (2001 ▶). A description of hydrogen bonding in terms of graph sets is given by Etter (1990 ▶) and Bernstein *et al.* (1995) ▶. For standard bond lengths as calculated using crystal structure data, see Allen *et al.* (2006 ▶).
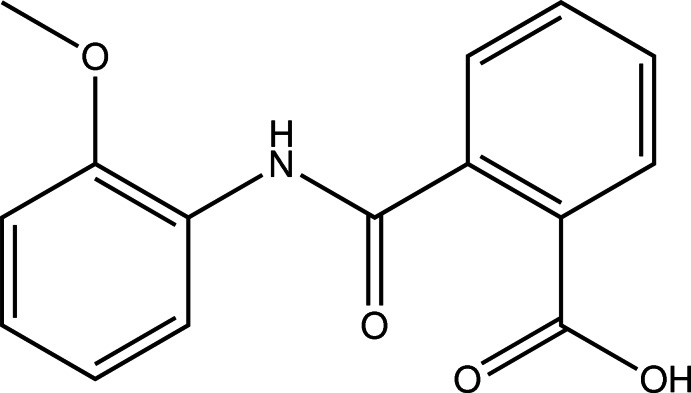


## Experimental

### 

#### Crystal data

C_15_H_13_NO_4_
*M* *_r_* = 271.26
Monoclinic, 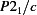

*a* = 9.038 (4) Å
*b* = 14.237 (5) Å
*c* = 10.405 (4) Å
β = 107.456 (5)°
*V* = 1277.2 (9) Å^3^
*Z* = 4
Mo *K*α radiation
μ = 0.10 mm^−1^
*T* = 120 K
0.53 × 0.18 × 0.09 mm

#### Data collection

Rigaku Saturn724+ diffractometer
Absorption correction: multi-scan (*ABSCOR*; Higashi, 1995 ▶) *T*_min_ = 0.978, *T*_max_ = 0.991
9056 measured reflections
2904 independent reflections
2687 reflections with *I* > 2σ(*I*)
*R*_int_ = 0.034

#### Refinement

*R*[*F* ^2^ > 2σ(*F* ^2^)] = 0.044
*wR*(*F* ^2^) = 0.111
*S* = 1.09
2904 reflections
233 parameters
All H-atom parameters refined
Δρ_max_ = 0.30 e Å^−3^
Δρ_min_ = −0.20 e Å^−3^

### 

Data collection: *CrystalClear-SM Expert* (Rigaku, 2009 ▶); cell refinement: *CrystalClear-SM Expert*; data reduction: *CrystalClear-SM Expert*; program(s) used to solve structure: *SHELXS97* (Sheldrick, 2008 ▶); program(s) used to refine structure: *SHELXL97* (Sheldrick, 2008 ▶); molecular graphics: *SHELXTL* (Sheldrick, 2008 ▶); software used to prepare material for publication: *WinGX* (Farrugia, 2012 ▶).

## Supplementary Material

Crystal structure: contains datablock(s) I, global. DOI: 10.1107/S1600536813013408/gw2134sup1.cif

Structure factors: contains datablock(s) I. DOI: 10.1107/S1600536813013408/gw2134Isup2.hkl

Supplementary material file. DOI: 10.1107/S1600536813013408/gw2134Isup3.cml

Additional supplementary materials:  crystallographic information; 3D view; checkCIF report

## Figures and Tables

**Table 1:** Hydrogen-bond geometry (Å, °)

*D*—H⋯*A*	*D*—H	H⋯*A*	*D*⋯*A*	*D*—H⋯*A*
O1—H1*O*⋯O3^i^	0.97 (3)	1.72 (3)	2.6837 (16)	175 (2)

Symmetry code: (i) 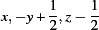.

**Fig. 1.**
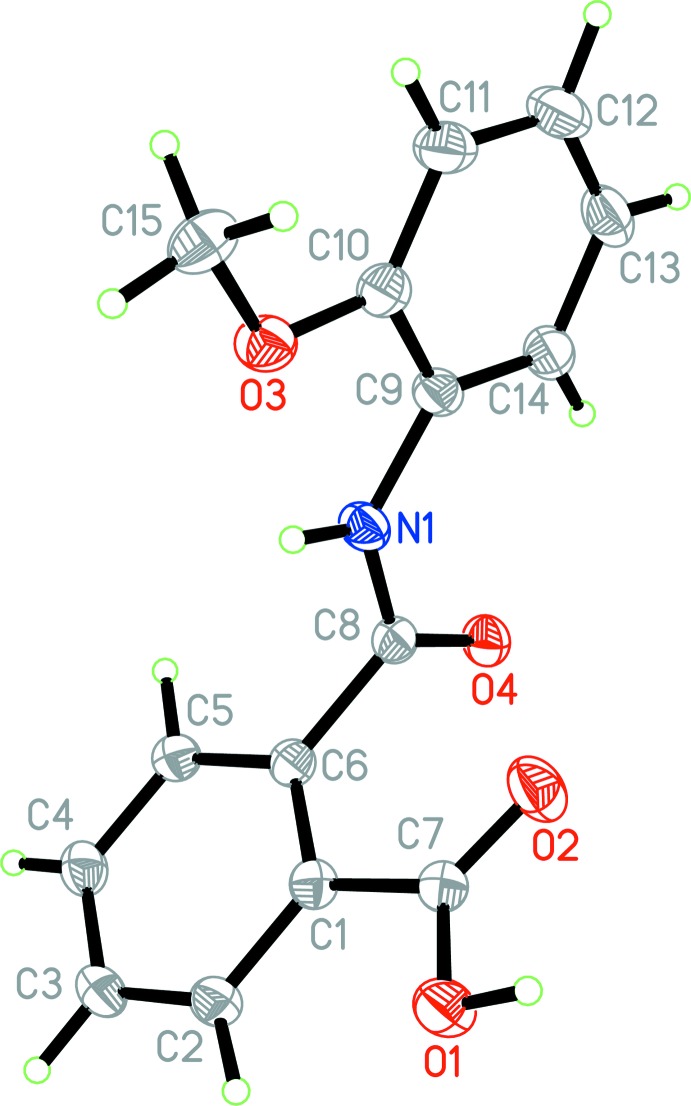
The asymmetric unit of the title compound with displacement ellipsoids drawn at the 50% probability level.

**Fig. 2.**
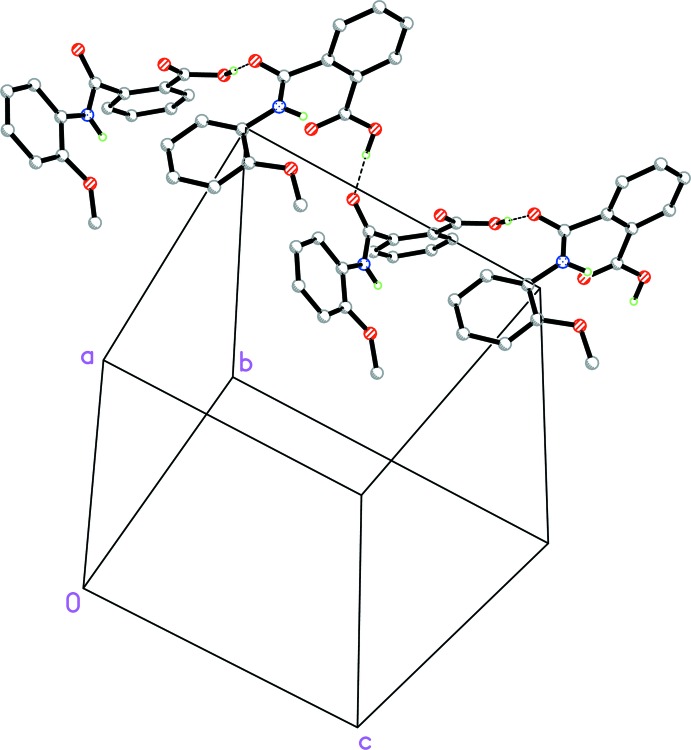
View of the C(7) hydrogen bond (dashed lines) motif in the [001] direction in the structure of the title compound. Carbon-bonded hydrogen atoms are omitted for clarity.

## supplementary crystallographic information

### Comment

Molecules of the title compound (Fig. 1.) exhibit a conformation wherein the interplanar angle between the two phenyl rings is observed to be 87.30 (5)°. The 2-methoxy-substituted ring is in almost perfect alignment with the amide moiety, the interplanar angle between the two being 3.23 (15)°. However, the twisted conformation is due to the almost 90° interplanar angle between the amide and the carboxy-substituted phenyl ring (89.81 (8)°). This conformation is similar to that observed in the structure of *N*-(2-phenyl)phthalamic acid (Smith *et al.* 1983; Bocelli *et al.* 1989). It should also be noted that a more planar conformation is observed where an intramolecular hydrogen bond can be formed between the hydrogen of the amide moiety and a hydrogen bond acceptor at the 2-position of the ring adjacent to the amide carbonyl, as is observed in *N*-(2-methoxyphenyl)-2-methoxybenzamide (Parra *et al.* 2001).

An intermolecular hydrogen bond is observed in the interaction between O1, in the carboxylic acid moiety of one molecule (acting as the hydrogen bond donor), and O3, the amide carbonyl of an adjacent molecule (which acts as the acceptor). This O1—H1O···O3 hydrogen bond (1.72 (3) Å) links the molecules along the crystallographic <001> direction (Fig. 2.) and, as a result, the molecules in the chain are related by the glide plane symmetry element along the *c* axis. It is worth noting that despite the presence of four oxygen atoms, all of which have the potential to act as hydrogen bond acceptors, the amide proton, H1N, does not form a hydrogen bond in this structure; a rare exception to Etter's first rule (Etter, 1990). In this case, a number of weak C—H···O hydrogen bonds form in preference, with the carbon atoms of the aromatic rings and their associated protons acting as the hydrogen bond donors.

Within the molecule itself, there are four distinct oxygen atom environments. The carboxylic acid oxygen atoms, O1 and O2, exhibit bond lengths to the carbon atom of the acid group within two standard deviations of the *International Tables for Crystallography* value (Allen *et al.* 2006) demonstrating distinct single and double bond character respectively. This suggests very little resonance-related charge seperation across the carboxylic acid group in this molecule. The same cannot be said of the amide moeity, however, as the carbonyl bond is longer in this case due to resonance between O3 and N1. This is a far from unexpected development as the C8—O3 bond length is close to the average for bonds of this type. There is no resonance form for this molecule associated with a change in the bond lengths to O4 and hence these bond lengths are also in agreement with the *International Tables for Crystallography* values.

### Experimental

*N*-(2-methoxyphenyl)phthalamic acid was obtained from Sigma-Aldrich. Crystals suitable for single-crystal X-ray crystallography were grown *via* evaporation of the solvent from a solution of the product in methanol.

### Refinement

H atoms were located from the Fourier difference map of electron density and refined freely. The most disagreeable reflections were omitted; three reflections exhibiting a Δ(F^2^) value greater than 5 su were removed.

### Crystal data

**Table:**

C_15_H_13_NO_4_	*F*(000) = 568
*M**_r_* = 271.26	*D*_x_ = 1.411 Mg m^−^^3^
Monoclinic, *P*2_1_/*c*	Mo *K*α radiation, λ = 0.71073 Å
Hall symbol: -P 2ybc	Cell parameters from 3461 reflections
*a* = 9.038 (4) Å	θ = 2.5–33.2°
*b* = 14.237 (5) Å	µ = 0.10 mm^−^^1^
*c* = 10.405 (4) Å	*T* = 120 K
β = 107.456 (5)°	Prism, colorless
*V* = 1277.2 (9) Å^3^	0.53 × 0.18 × 0.09 mm
*Z* = 4	

### Data collection

**Table:**

Rigaku Saturn724+ diffractometer	2904 independent reflections
Radiation source: Sealed Tube	2687 reflections with *I* > 2σ(*I*)
Graphite monochromator	*R*_int_ = 0.034
Detector resolution: 28.5714 pixels mm^-1^	θ_max_ = 27.5°, θ_min_ = 3.8°
profile data from ω–scans	*h* = −11→11
Absorption correction: multi-scan (*ABSCOR*; Higashi, 1995)	*k* = −18→18
*T*_min_ = 0.978, *T*_max_ = 0.991	*l* = −8→13
9056 measured reflections	

### Refinement

**Table:**

Refinement on *F*^2^	Primary atom site location: structure-invariant direct methods
Least-squares matrix: full	Secondary atom site location: difference Fourier map
*R*[*F*^2^ > 2σ(*F*^2^)] = 0.044	Hydrogen site location: inferred from neighbouring sites
*wR*(*F*^2^) = 0.111	All H-atom parameters refined
*S* = 1.09	*w* = 1/[σ^2^(*F*_o_^2^) + (0.0518*P*)^2^ + 0.4728*P*] where *P* = (*F*_o_^2^ + 2*F*_c_^2^)/3
2904 reflections	(Δ/σ)_max_ < 0.001
233 parameters	Δρ_max_ = 0.30 e Å^−^^3^
0 restraints	Δρ_min_ = −0.20 e Å^−^^3^

### Special details

**Table:**

Geometry. All s.u.'s (except the s.u. in the dihedral angle between two l.s. planes) are estimated using the full covariance matrix. The cell s.u.'s are taken into account individually in the estimation of s.u.'s in distances, angles and torsion angles; correlations between s.u.'s in cell parameters are only used when they are defined by crystal symmetry. An approximate (isotropic) treatment of cell s.u.'s is used for estimating s.u.'s involving l.s. planes.
Refinement. Refinement of *F*^2^ against ALL reflections. The weighted *R*-factor *wR* and goodness of fit *S* are based on *F*^2^, conventional *R*-factors *R* are based on *F*, with *F* set to zero for negative *F*^2^. The threshold expression of *F*^2^ > 2σ(*F*^2^) is used only for calculating *R*-factors(gt) *etc*. and is not relevant to the choice of reflections for refinement. *R*-factors based on *F*^2^ are statistically about twice as large as those based on *F*, and *R*- factors based on ALL data will be even larger.

### Fractional atomic coordinates and isotropic or equivalent isotropic displacement parameters (Å^2^)

**Table:**

	*x*	*y*	*z*	*U*_iso_*/*U*_eq_	
C1	0.38628 (15)	0.07876 (9)	0.59059 (13)	0.0212 (3)	
C2	0.45227 (14)	0.03553 (9)	0.71557 (13)	0.0204 (3)	
C3	0.42309 (16)	−0.05935 (9)	0.73260 (14)	0.0230 (3)	
C4	0.32498 (17)	−0.10968 (9)	0.62659 (14)	0.0258 (3)	
C5	0.25493 (17)	−0.06637 (10)	0.50393 (14)	0.0261 (3)	
C6	0.28625 (16)	0.02755 (10)	0.48532 (13)	0.0239 (3)	
C7	0.42837 (15)	0.17822 (9)	0.57121 (13)	0.0221 (3)	
C8	0.55286 (15)	0.08745 (9)	0.83745 (13)	0.0209 (3)	
C9	0.83209 (15)	0.12208 (9)	0.95264 (13)	0.0228 (3)	
C10	0.97791 (16)	0.11002 (9)	0.93125 (14)	0.0243 (3)	
C11	1.11088 (17)	0.14262 (10)	1.02478 (15)	0.0299 (3)	
C12	1.09965 (19)	0.18763 (11)	1.14052 (16)	0.0356 (4)	
C13	0.95702 (19)	0.20079 (10)	1.16090 (15)	0.0325 (3)	
C14	0.82184 (17)	0.16842 (10)	1.06717 (14)	0.0264 (3)	
C15	1.11647 (18)	0.05563 (12)	0.78206 (18)	0.0324 (3)	
N1	0.70557 (13)	0.08237 (8)	0.85182 (12)	0.0237 (2)	
O1	0.37604 (13)	0.20728 (7)	0.44416 (10)	0.0297 (2)	
O2	0.50580 (14)	0.22662 (7)	0.66190 (11)	0.0353 (3)	
O3	0.49670 (11)	0.12378 (7)	0.91995 (9)	0.0251 (2)	
O4	0.97329 (11)	0.06458 (7)	0.81392 (10)	0.0278 (2)	
H1N	0.731 (2)	0.0563 (13)	0.7835 (19)	0.034 (5)*	
H1O	0.425 (3)	0.2672 (18)	0.440 (2)	0.062 (7)*	
H3	0.481 (2)	−0.0877 (12)	0.8217 (19)	0.032 (4)*	
H4	0.304 (2)	−0.1725 (13)	0.6414 (17)	0.028 (4)*	
H5	0.185 (2)	−0.1009 (13)	0.4320 (18)	0.032 (4)*	
H6	0.2416 (18)	0.0616 (11)	0.4002 (16)	0.019 (4)*	
H11	1.209 (2)	0.1333 (13)	1.0088 (18)	0.037 (5)*	
H12	1.194 (3)	0.2081 (15)	1.203 (2)	0.048 (6)*	
H13	0.944 (2)	0.2302 (13)	1.2380 (19)	0.035 (5)*	
H14	0.728 (2)	0.1778 (12)	1.0829 (17)	0.026 (4)*	
H15A	1.088 (2)	0.0212 (14)	0.694 (2)	0.044 (5)*	
H15B	1.158 (2)	0.1163 (14)	0.7720 (19)	0.039 (5)*	
H15C	1.192 (2)	0.0199 (13)	0.8516 (18)	0.033 (5)*	

### Atomic displacement parameters (Å^2^)

**Table:**

	*U*^11^	*U*^22^	*U*^33^	*U*^12^	*U*^13^	*U*^23^
C1	0.0216 (6)	0.0207 (6)	0.0216 (6)	0.0001 (5)	0.0070 (5)	−0.0006 (5)
C2	0.0187 (6)	0.0220 (6)	0.0215 (6)	0.0009 (5)	0.0074 (5)	−0.0014 (5)
C3	0.0226 (6)	0.0225 (6)	0.0230 (6)	0.0005 (5)	0.0051 (5)	0.0006 (5)
C4	0.0277 (7)	0.0205 (6)	0.0294 (7)	−0.0033 (5)	0.0090 (6)	−0.0023 (5)
C5	0.0264 (7)	0.0257 (6)	0.0243 (7)	−0.0045 (5)	0.0050 (5)	−0.0058 (5)
C6	0.0245 (7)	0.0259 (6)	0.0202 (6)	−0.0001 (5)	0.0051 (5)	−0.0014 (5)
C7	0.0229 (6)	0.0211 (6)	0.0217 (6)	0.0006 (5)	0.0056 (5)	−0.0003 (5)
C8	0.0227 (6)	0.0185 (5)	0.0202 (6)	0.0010 (5)	0.0047 (5)	0.0006 (5)
C9	0.0242 (7)	0.0199 (6)	0.0211 (6)	−0.0019 (5)	0.0020 (5)	0.0019 (5)
C10	0.0263 (7)	0.0210 (6)	0.0240 (6)	−0.0002 (5)	0.0051 (5)	0.0038 (5)
C11	0.0236 (7)	0.0285 (7)	0.0333 (8)	−0.0028 (5)	0.0020 (6)	0.0045 (6)
C12	0.0324 (8)	0.0316 (7)	0.0329 (8)	−0.0074 (6)	−0.0052 (6)	0.0002 (6)
C13	0.0429 (9)	0.0263 (7)	0.0241 (7)	−0.0045 (6)	0.0034 (6)	−0.0052 (6)
C14	0.0294 (7)	0.0239 (6)	0.0246 (7)	−0.0011 (5)	0.0059 (6)	−0.0009 (5)
C15	0.0252 (7)	0.0351 (8)	0.0393 (9)	0.0056 (6)	0.0134 (6)	0.0062 (7)
N1	0.0217 (6)	0.0283 (6)	0.0207 (6)	−0.0011 (4)	0.0058 (4)	−0.0063 (4)
O1	0.0356 (6)	0.0259 (5)	0.0240 (5)	−0.0053 (4)	0.0036 (4)	0.0033 (4)
O2	0.0497 (7)	0.0240 (5)	0.0263 (5)	−0.0099 (4)	0.0026 (5)	0.0002 (4)
O3	0.0253 (5)	0.0269 (5)	0.0238 (5)	0.0014 (4)	0.0083 (4)	−0.0054 (4)
O4	0.0225 (5)	0.0342 (5)	0.0277 (5)	0.0002 (4)	0.0091 (4)	−0.0024 (4)

### Geometric parameters (Å, º)

**Table:**

C1—C6	1.3980 (19)	C9—C10	1.411 (2)
C1—C2	1.4001 (18)	C9—N1	1.4171 (17)
C1—C7	1.4955 (18)	C10—O4	1.3711 (17)
C2—C3	1.3977 (19)	C10—C11	1.380 (2)
C2—C8	1.5131 (18)	C11—C12	1.394 (2)
C3—C4	1.3891 (19)	C11—H11	0.961 (19)
C3—H3	1.002 (18)	C12—C13	1.381 (2)
C4—C5	1.387 (2)	C12—H12	0.95 (2)
C4—H4	0.937 (18)	C13—C14	1.393 (2)
C5—C6	1.392 (2)	C13—H13	0.942 (19)
C5—H5	0.956 (19)	C14—H14	0.920 (17)
C6—H6	0.984 (16)	C15—O4	1.4346 (18)
C7—O2	1.2079 (17)	C15—H15A	1.00 (2)
C7—O1	1.3293 (17)	C15—H15B	0.96 (2)
C8—O3	1.2343 (16)	C15—H15C	0.975 (19)
C8—N1	1.3449 (19)	N1—H1N	0.889 (19)
C9—C14	1.389 (2)	O1—H1O	0.97 (3)
			
C6—C1—C2	119.58 (12)	O4—C10—C11	125.02 (13)
C6—C1—C7	121.26 (12)	O4—C10—C9	114.67 (12)
C2—C1—C7	119.13 (12)	C11—C10—C9	120.31 (13)
C3—C2—C1	119.91 (12)	C10—C11—C12	119.33 (14)
C3—C2—C8	117.04 (11)	C10—C11—H11	119.1 (11)
C1—C2—C8	123.03 (12)	C12—C11—H11	121.6 (12)
C4—C3—C2	119.84 (12)	C13—C12—C11	120.55 (14)
C4—C3—H3	123.9 (10)	C13—C12—H12	122.4 (13)
C2—C3—H3	116.2 (10)	C11—C12—H12	117.1 (13)
C5—C4—C3	120.51 (13)	C12—C13—C14	120.71 (15)
C5—C4—H4	121.1 (11)	C12—C13—H13	123.3 (11)
C3—C4—H4	118.3 (11)	C14—C13—H13	115.9 (11)
C4—C5—C6	119.94 (13)	C9—C14—C13	119.17 (14)
C4—C5—H5	120.2 (11)	C9—C14—H14	121.5 (11)
C6—C5—H5	119.9 (11)	C13—C14—H14	119.3 (11)
C5—C6—C1	120.17 (13)	O4—C15—H15A	104.6 (12)
C5—C6—H6	123.6 (9)	O4—C15—H15B	110.8 (11)
C1—C6—H6	116.2 (9)	H15A—C15—H15B	110.0 (16)
O2—C7—O1	123.32 (12)	O4—C15—H15C	110.7 (11)
O2—C7—C1	123.10 (12)	H15A—C15—H15C	110.7 (16)
O1—C7—C1	113.56 (11)	H15B—C15—H15C	110.0 (16)
O3—C8—N1	124.58 (12)	C8—N1—C9	129.36 (12)
O3—C8—C2	121.24 (12)	C8—N1—H1N	115.7 (12)
N1—C8—C2	113.92 (11)	C9—N1—H1N	114.5 (12)
C14—C9—C10	119.92 (13)	C7—O1—H1O	106.7 (14)
C14—C9—N1	125.29 (13)	C10—O4—C15	117.33 (12)
C10—C9—N1	114.78 (12)		
			
C6—C1—C2—C3	2.71 (19)	C1—C2—C8—N1	−93.21 (15)
C7—C1—C2—C3	−175.39 (12)	C14—C9—C10—O4	−179.05 (12)
C6—C1—C2—C8	−175.73 (12)	N1—C9—C10—O4	2.14 (16)
C7—C1—C2—C8	6.17 (18)	C14—C9—C10—C11	1.1 (2)
C1—C2—C3—C4	−1.92 (19)	N1—C9—C10—C11	−177.70 (12)
C8—C2—C3—C4	176.61 (12)	O4—C10—C11—C12	−179.85 (13)
C2—C3—C4—C5	−0.3 (2)	C9—C10—C11—C12	0.0 (2)
C3—C4—C5—C6	1.8 (2)	C10—C11—C12—C13	−0.9 (2)
C4—C5—C6—C1	−1.0 (2)	C11—C12—C13—C14	0.8 (2)
C2—C1—C6—C5	−1.3 (2)	C10—C9—C14—C13	−1.2 (2)
C7—C1—C6—C5	176.80 (12)	N1—C9—C14—C13	177.44 (13)
C6—C1—C7—O2	174.83 (14)	C12—C13—C14—C9	0.3 (2)
C2—C1—C7—O2	−7.1 (2)	O3—C8—N1—C9	−5.9 (2)
C6—C1—C7—O1	−6.92 (18)	C2—C8—N1—C9	179.87 (12)
C2—C1—C7—O1	171.15 (11)	C14—C9—N1—C8	6.6 (2)
C3—C2—C8—O3	−86.11 (16)	C10—C9—N1—C8	−174.66 (13)
C1—C2—C8—O3	92.37 (16)	C11—C10—O4—C15	−4.18 (19)
C3—C2—C8—N1	88.31 (14)	C9—C10—O4—C15	176.00 (12)

### Hydrogen-bond geometry (Å, º)

**Table:**

*D*—H···*A*	*D*—H	H···*A*	*D*···*A*	*D*—H···*A*
O1—H1*O*···O3^i^	0.97 (3)	1.72 (3)	2.6837 (16)	175 (2)

Symmetry code: (i) *x*, −*y*+1/2, *z*−1/2.
